# Analysis of Changes in Flavor Profile and Bacterial Succession During Pork Fermentation Using Multi-Omics-Based Analysis

**DOI:** 10.3390/foods14213804

**Published:** 2025-11-06

**Authors:** Yuyan Ma, Qiuyu Lan, Chenshuo Wang, Luca Laghi, Chenglin Zhu, Gianfranco Picone

**Affiliations:** 1 College of Pharmacy and Food, Southwest Minzu University, Chengdu 610041, China; 2 Modern Industrial College of Traditional Chinese Medicine and Health, Zhejiang Lishui Service Platform for Technological Innovations in Traditional Chinese Medicine Industry, Lishui Institute of Traditional Chinese Medicine, Lishui University, Lishui 323000, China; 3 Department of Agricultural and Food Sciences, University of Bologna, 47521 Cesena, Italy

**Keywords:** sour meat, fermentation, RNA sequencing analysis, aroma compounds, bacterial community

## Abstract

Sour meat, a traditional fermented meat product, derives its unique attributes from the flavors developed during the fermentation process. This study systematically investigated the dynamic changes in volatile compounds and bacterial succession in pork sour meat during fermentation (0, 15, 30, and 45 days) using a combination of an electric nose (E-nose), an electric tongue (E-tongue), gas chromatography–ion mobility spectrometry (GC-IMS), gas chromatography–mass spectrometry (GC-MS), and 16S rRNA amplicon sequencing. The results showed that the E-nose and E-tongue effectively distinguished samples across fermentation stages. The pork sour meat was analyzed using GC-IMS and GC-MS, which identified 39 and 81 volatile compounds (VOCs), respectively, primarily esters, alcohols, and aldehydes, with esters being most abundant after 45 days of fermentation. A total of 18 and 25 volatile compounds, respectively, were identified by GC-IMS and GC-MS as differential VOCs (*p* < 0.05, VIP > 1) of the pork sour meat. *α*-diversity increased in both species’ richness and diversity over the course of fermentation, while *β*-diversity analysis further differentiated samples across stages. *Firmicutes* dominated the bacterial community, with *Staphylococcus*, *Lactobacillus*, and *Weissella* as the main genera. Pearson correlation analysis revealed distinct associations between bacteria and volatiles: *Staphylococcus* was positively associated with butyl acetate-D, ethyl acetate, isoamyl acetate, dihydroactinidiolide, and *(E)*-2-heptenal, while *Lactobacillus* and *Weissella* were positively associated with acetic acid. Additionally, *Weissella* showed positive correlations with eight volatile compounds: acetic acid, nonanal, benzyl alcohol, ethyl crotonate, isoamyl acetate, dihydroactinidiolide, octanal, and ethyl acetate. This study provides a comprehensive understanding of volatile compound evolution and bacterial succession in pork sour meat, thereby offering a scientific basis for understanding and regulating its flavor quality.

## 1. Introduction

Sour meat is a traditional fermented meat product widely consumed in the Hunan, Guizhou, and Guangxi provinces of China. It is prepared by mixing fresh pork slices with salt, glutinous rice, and seasonings such as chili powder and pepper, then sealed and naturally fermented [1]. During fermentation, sour meat generates esters, alcohols, acids, amino acids, and other compounds that collectively enhance its nutritional value and flavor [2]. The characteristic flavor develops through complex bacterial interactions and associated biochemical transformations (protein degradation, lipid oxidation, and carbohydrate metabolism) [3]. However, inconsistency in flavor among different batches remains a major obstacle to the standardized production and wider acceptance of sour meat. Ensuring consistent flavor quality therefore remains an urgent challenge. Understanding the changes in flavor and bacterial succession during fermentation is essential for achieving standardized, high-quality sour meat products.

Flavor, a key determinant of consumer preference for fermented foods [4], is typically analyzed using GC-MS due to its powerful qualitative and quantitative capabilities [5]. Complementing GC-MS, GC-IMS is an emerging analytical technique offering high precision, good selectivity, and low detection limits. Both techniques enable intuitive visualization of differences and changes in food volatile compounds [6]. Integrating these methods with E-nose and E-tongue analyses provides a comprehensive evaluation of food flavor profiles. In contrast to traditional sensory methods that are prone to fatigue and bias, E-noses and E-tongues objectively simulate human smell and taste, ensuring reproducible and analytically robust results. They consist of sensors designed to obtain a holistic fingerprint of the molecular features that produce complex odors and tastes [7]. These methods offer several advantages, including rapid response, ease of use, reliability, and accuracy [8].

Bacterial metabolism plays a central role in flavor formation during fermentation. *Staphylococcus*, *Lactobacillus*, and *Micrococcusare* key members of the microbial community in fermented meat products [9], generating diverse metabolites through metabolic pathways such as lactic acid, alcoholic fermentation, amino acid catabolism, and esterification [10,11,12]. For example, *Lactobacillus* converts sugars into lactic acid during fermentation, forming the basis for subsequent flavor compound development. Advancements in multi-omics methodologies have further expanded our understanding of flavor evolution and microbial succession in fermented foods such as Baijiu [13], kimchi [14], sausage [15], ham [16], and bamboo shoots [17]. Recent studies on fermented pork sour meat have employed GC-MS and sequencing to map flavor profiles and bacterial dynamics [18,19]. However, integrated analyses combining the E-nose, E-tongue, GC-IMS, and GC-MS techniques remain limited.

This study intended to comprehensively analyze the changes in and correlations between bacterial communities and flavor substances of pork sour meat in different fermentation stages by combining intelligent senses (E-nose, E-tongue), GC-IMS, GC-MS, and 16S rRNA and to provide a more comprehensive overview of the flavor profile of sour meat by revealing the intrinsic connection between these changes, which would provide a further reference for improving the flavor quality of sour meat.

## 2. Materials and Methods

### 2.1. Sample Preparation

Fresh pork was purchased from a local market in Chengdu, Sichuan province, China. Following the traditional artisanal procedure used in Yunnan Province, fresh pork was cut into small pieces (3 cm × 5 cm × 0.6 cm), mixed with 6% salt (*w*/*w*), and cured at 4 °C for 2 h. Subsequently, 15% chili powder (*w*/*w*) was added, together with 25% fried and coarsely ground glutinous rice (*w*/*w*) and 25% fried and steamed glutinous rice (*w*/*w*). Finally, the mixtures were transferred into sealed containers and fermented at 20 ± 2 °C for 0, 15, 30, and 45 days, designated as P0, P15, P30, and P45, respectively. Each fermentation jar contained 200 g of sample, with three biological replicates per group.

### 2.2. E-Nose Analysis

An E-nose (FOX 3000, Alpha MOS, Toulouse, France) was used to evaluate the overall volatile profile of sour meat samples following Zhao et al. [20]. The system comprises three metal oxide sensor chambers and 18 oxide gas sensors (Table S1). For each analysis, a 0.25 g sour meat sample portion was placed into a 10 mL headspace vial. The samples were incubated at a constant temperature of 70 °C for 5 min to allow complete release of VOCs. The prepared sample was manually injected into the E-nose system, with the measurement process strictly controlled to ensure completion within 120 s. After each measurement, the rinsing phase lasted 240 s, with the sensor array thoroughly cleaned and reset to prevent cross-contamination between samples. For each sour meat sample, five replicate observations were performed, and three stable datasets were selected based on the following criterion: the two datasets showing the greatest deviation from the average response value were excluded, ensuring that the remaining datasets had minimal deviation and were representative of the sample’s volatile profile.

### 2.3. E-Tongue Analysis

An *α*-ASTREE E-tongue system (Alpha MOS, France) was employed in this study to assess the overall taste profile of sour meat samples following the methodology described by Wang et al. [21]. Prior to measurement, the sensors were immersed in a beaker for passive activation (1 h) and active activation (0.5 h) in 0.01 mol/L HCl solution followed by a calibration sequence under strict parameters. The sensors were diagnosed using a solution of 0.01 mol/L HCl, MSG, and NaCl to ensure that they were in a stable working condition. The taste analysis instrument consists primarily of two reference electrodes (PKS and CPS) and five liquid cross-sensitive electrodes (ANS, CTS, NMS, AHS, SCS), which respond to sweetness, saltiness, umami, sourness, and bitterness, respectively. A total of 20 g of the sample was chopped and mixed with 200 mL of deionized water. The mixed solution was centrifuged at 2265× *g* for 10 min at 4 °C to obtain the sample (80 mL) for E-tongue analysis. The deionized water was used as the washing solution, and the main parameters of the instrument were as follows: acquisition time, 120 s; stirring rate, 60 rpm; washing time, 30 s. Each sample was analyzed in technical triplicate.

### 2.4. GC-IMS Analysis

GC-IMS (Flavorspec^®^, G.A.S. Instrument, Dortmund, Germany) was applied to analyze the volatile compounds in pork sour meat at different fermentation stages. Referring to the method of Zhao et al. [20], 0.25 g of sample was equilibrated in headspace vials (50 °C, 10 min) and extracted (65 °C) using an automatic headspace sampling system. The experimental parameters of IMS were set as follows: the column was MXT-WAX (30 m × 0.53 mm × 1 µm) (Restek, Bellefonte, PA, USA), and the flow rates of the carrier gas (N2) were 2 mL/min for 5 min, 10 mL/min for 10 min, 15 mL/min for 5 min, 50 mL/min for 10 min, and 100 mL/min for 10 min. The retention index (RI) of each compound was calculated using n-alkanes (C4-C9) as external references. The volatile compounds were identified by comparing the retention index and drift time with the GC-IMS library. Volatile compound quantification was based on the peak signal intensity. The values were semi-quantitative as no internal standards were applied. Using the Laboratory Analytical Viewer, Reporter, and Gallery Plot supported by the GC-IMS instrument, three-dimensional and topographic subtraction maps and gallery plots of the volatile compounds were constructed. Each sample was analyzed in technical triplicate.

### 2.5. GC-MS Analysis

The volatile components from the pork sour meat at different fermentation stages were extracted using headspace solid-phase microextraction (HS-SPME) with a 50/30 μm DVB/CAR/PDMS fiber. Subsequently, the analysis was performed on a GC-MS system (Thermo Fisher Scientific, Waltham, MA, USA) equipped with a TG-WAXMS B column (30 m × 0.25 mm × 0.25 µm) and a Triplus auto-sampler (Thermo Fisher Scientific, Waltham, MA, USA). A 5 g sample of pork was put into a 20 mL headspace bottle and sealed for 30 min at 50 °C. The volatile compounds were extracted utilizing HS-SPME, followed by GC-MS analysis, and each sample was detected three times. The injection port temperature was set to 230 °C and held for 15 min. The carrier gas was helium (≥99.999% purity) without a split mode. The initial oven temperature was set to 40 °C and held for 3 min. Then the temperature was increased at a rate of 5 °C/min to 210 °C and held for 5 min. Seventy electron volts of electron ionization with an ion source temperature of 250 °C and a 40–500 *m*/*z* scanning range were applied to obtain the mass spectrum. Volatile compounds were identified with the help of the NIST 11 library. Only values for the matching degree of molecules above 800 were reported. The classical area normalization method was applied, in which the peak area percentage of each volatile compound was used to represent its relative abundance within the sample.

### 2.6. Determination of Bacterial Community

Under aseptic conditions, a 0.25 g sample was taken from the pork sour meat at different fermentation stages, immediately placed in a sterile cryovial, rapidly frozen in liquid nitrogen, and stored at −80 °C. According to the manufacturer’s instructions, bacterial community genomic DNA was extracted from pork sour meat at different fermentation stages using the E.Z.N.A.^®^ soil DNA kit (Omega Biotek (version number: 6.0), Norcross, GA, USA). The hypervariable V3-V4 region of the bacterial 16S rRNA gene was amplified with the primers 338F (5′-ACTCCTACGGGAGGCAGCAG-3′) and 806R (5′-GGACTACHVGGGTWTCTAAT-3′) by A T100 Thermal Cycler, BIO-RAD PCR (Hercules, CA, USA). Purified amplicons were pooled in equimolar and paired-end sequences on an Illumina MiSeq PE300/PE250 platform (Illumina, San Diego, CA, USA), according to the standard protocols of Majorbio Bio-Pharm Technology Co., Ltd. (Shanghai, China). The raw 16S rRNA gene sequencing reads were demultiplexed, quality-filtered by fastp version 0.20.0, and merged by FLASH version 1.2.7. The optimized sequences obtained by quality-filtering and merging were further processed by DADA2, and the PCR amplification or sequencing errors existing in the optimized sequence were removed to obtain the real sequence in formation (Amplicon Sequence Variants, ASVs) in the samples [22]. Each sample was analyzed in technical triplicate.

### 2.7. Statistical Analysis

The statistical analysis was conducted using the R (4.4.1 version) computational language. Prior to conducting univariate analyses, the data distribution was normalized according to Box and Cox [23]. ANOVA was employed to identify significant differences among groups, followed by Tukey’s HSD test (*p* < 0.05). Following the methodology of our previous study [24], robust principal component analysis (rPCA) models were established based on the average values of the E-nose and E-tongue sensors, as well as the peak signal intensities of the molecules, respectively. For each rPCA model, a score plot and a Pearson correlation plot of the loadings were generated to elucidate the structure of the data and uncover the relationships between variables and model components. The principal component analysis (PCA) and partial least-squares discriminant analysis (PLS-DA) were set up through an online data mining tool, namely MetaboAnalyst 5.0 (https://www.metaboanalyst.ca, accessed on 15 May 2025). PLS-DA model validation (7-fold cross-validation and 200-permutation tests) was performed using SIMCA software (SIMCA 14.1). Correlation analysis was performed using online tools (cloud.metware.cn, accessed on 5 October 2024). Furthermore, diversity analysis and taxonomic composition analysis were performed on the microbiome data using https://www.majorbio.com/ (accessed on 15 May 2025).

## 3. Results

### 3.1. Intelligent Senses

#### 3.1.1. E-Nose Analysis

**Figure 1:**
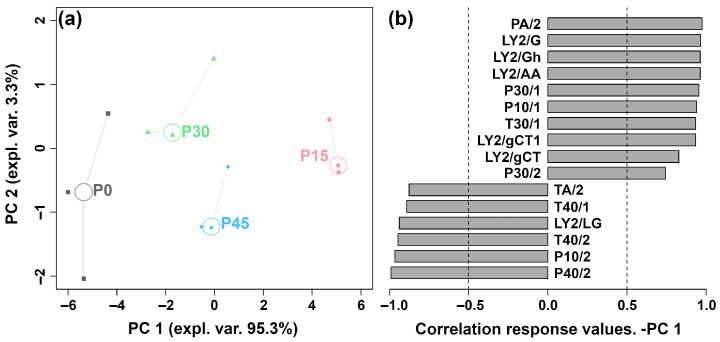
The robust principal component analysis (rPCA) model was constructed using E-nose sensor response values. (**a**) A score plot displaying the distribution of samples at different fermentation stages (P0, P15, P30, P45). (**b**) A Pearson correlation loading plot.

The E-nose system, equipped with 18 sensors, was employed to identify pork sour meat during fermentation and assess their overall aroma profiles. Responses from 16 sensors differed among the four groups. To discriminate the aroma profiles at different fermentation stages, an rPCA model was constructed based on the E-nose sensor responses, as shown in Figure 1.

As illustrated in Figure 1a, PC 1 and PC 2 cumulatively accounted for 98.6% of the total variance, explaining most of the data variance and highlighting group differences. Along the PC 1 axis, the P30 and P45 groups are positioned closely, indicating similar aroma profiles between pork sour meat samples fermented for 30 and 45 days. The P15 group is clearly separated from the P0, P30, and P45 groups. In Figure 1b, P15 is characterized by higher responses on sensors sensitive to nitrogen-containing compounds (e.g., ammonia, organic amines), alcohols, hydrocarbons, and organic solvents (PA/2, LY2/G, LY2/Gh, LY2/AA, P30/1, P10/1, T30/1, LY2/gCT1, LY2/gCT, and P30/2), and lower responses on sensors sensitive to aldehydes, chlorinated compounds, and oxidizing gases (TA/2, T40/1, LY2/LG, T40/2, P10/2, and P40/2).

#### 3.1.2. E-Tongue Analysis

**Figure 2:**
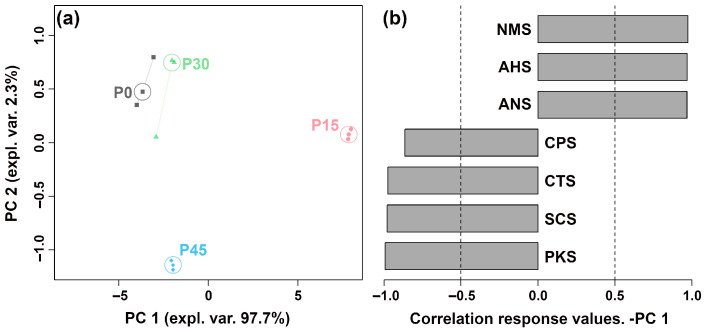
The robust principal component analysis (rPCA) model was constructed using E-tongue sensor response values. (**a**) A score plot displaying the distribution of samples across different fermentation stages (P0, P15, P30, P45). (**b**) A Pearson correlation loading plot.

The E-tongue system, equipped with seven sensors, was used to differentiate pork sour meat at different fermentation stages and evaluate their overall taste profiles. The sensors produced distinct responses across the four groups. To provide an overview of sensor trends, the response values were used to construct an rPCA model (Figure 2). As shown in Figure 2a, PC 1 explained 97.9% of the total variance, capturing the differences in taste characteristics among the groups. The loading analysis in Figure 2b indicates that P15 showed higher responses on the NMS, ANS, and AHS sensors than the other groups.

### 3.2. Flavor Profiles

#### 3.2.1. Flavor Characterization

**Figure 3:**
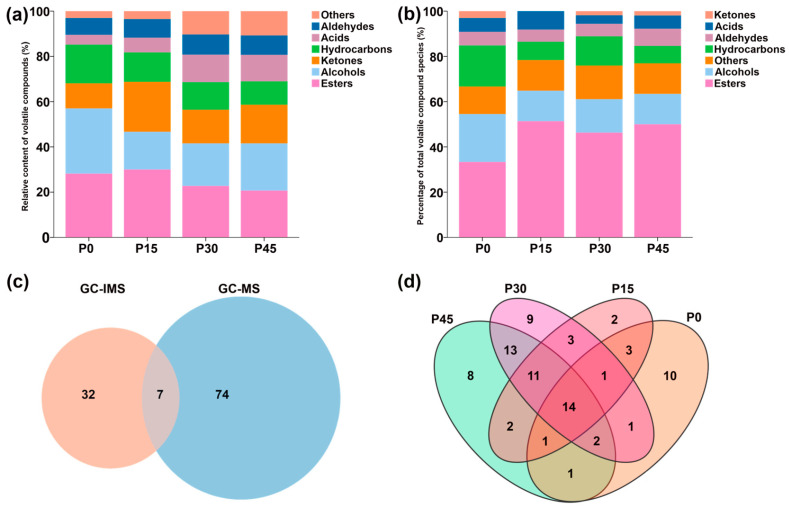
Aroma profiles of pork sour meat at different fermentation stages (P0, P15, P30, P45) as characterized by gas chromatography–ion mobility spectrometry (GC-IMS) (**a**) and gas chromatography–mass spectrometry (GC-MS) (**b**), respectively. (**c**) Venn diagram of the number of volatile compounds characterized by GC-IMS and GC-MS. (**d**) Venn diagram of the number of unique and shared volatile compounds in different sample groups characterized by GC-MS.

GC-IMS analysis identified 39 volatile compounds in pork sour meat across different fermentation stages, categorized as alcohols (12), hydrocarbons (9), esters (6), aldehydes (5), acids (1), ketones (4), and others (2) (Table S2). The relative abundances of these compounds differed among groups (Figure 3a). GC-MS analysis identified 81 volatile compounds, including esters (29), alcohols (13), aldehydes (4), hydrocarbons (13), ketones (2), acids (8), and others (12) (Table S3). Specifically, 33, 37, 54, and 52 volatile compounds were detected in the P0, P15, P30, and P45 groups, respectively, and the relative percentages varied among groups (Figure 3b). Figure 3c shows that seven compounds were detected by both techniques, whereas 32 and 74 compounds were uniquely identified by GC-IMS and GC-MS, respectively. Fourteen volatile compounds were common to all fermentation stages, indicating that esters were the predominant class among them (Table S3). The P30 and P45 groups had the highest percentage of esters among the shared compounds. Moreover, the unique compounds 10 (ethyl 2-ethylhexanoate, 2-ethylhexanol, 1-octanol, furfuryl alcohol, aromadendrene, limonene, 2-heptanone, lauric acid, arachidic acid, and 1,1-diethoxyethane), 2 (decane, 2,4,6-trimethyl-, and 2-methyl-2-pentenoic acid), 9 (hexyl hexanoate, 1-nonanol, 1-hexadecanol, 2-methyl-, hexadecane, 2,6,10-trimethyldodecane, 2,6,10-trimethyltetradecane, 5-aminovaleric acid, *D*-mannose, and formohydrazide), and 8 (ethyl laurate, 4-isopropylbenzyl alcohol, 4-methyl-1-pentanol, 2,6,7-trimethyl-decane, acetic acid, methoxyacetic acid, 2,4,5-trimethyl-1,3-dioxolane, and *β*-lactose) were detected in the P0, P15, P30, and P45 groups, respectively.

#### 3.2.2. GC-IMS Analysis

**Figure 4:**
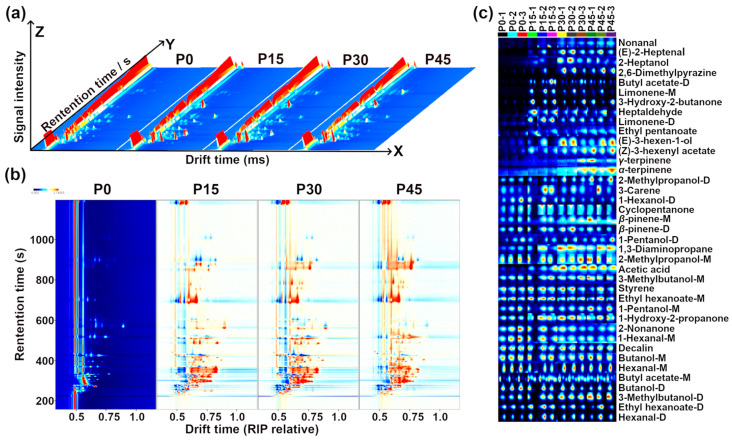
Gas chromatography–ion mobility spectrometry (GC-IMS) observation results for pork sour meat at different fermentation stages (P0, P15, P30, P45). (**a**) Three-dimensional topographic map, (**b**) topographic subtraction map, and (**c**) gallery plots. In the topographic subtraction map, the spectrum of the P0 group is used as a reference, and the corresponding spectra from the P15, P30, and P45 groups represent the differences from the P0 group. In the gallery plots, red and blue colors highlight over- and under-expressed components, respectively.

The result of GC-IMS information regarding the volatile compounds in pork sour meat is illustrated in Figure 4.

Three-dimensional spectra of volatile compounds in pork sour meat at different fermentation stages are shown in Figure 4a. The results indicate that signal intensities varied significantly across fermentation stages, with changes in compound types. Signal intensity was used to indicate the relative abundance of volatile compounds. To compare differences among samples, the spectrograms of the raw samples were used as a reference, and a sample difference plot was obtained by subtracting the reference spectra from the other three samples. In Figure 4b, the subtracted background appears white when relative abundance matches the reference, blue when it is below the reference, and red when it is above it [25]. Point-by-point comparison revealed that volatile compounds were mainly detected at retention times of 330–900 s and drift times of 1.1–1.8 ms. The types and relative abundances of volatile compounds at different fermentation stages are shown in Figure 4c.

**Figure 5:**
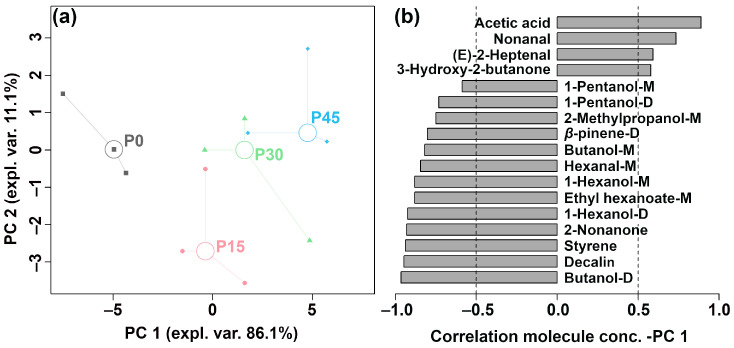
The robust principal component analysis (rPCA) model was established based on the relative abundance of differential volatile compounds according to gas chromatography-ion mobility spectrometry (GC-IMS). (**a**) A score plot displaying the distribution trends of samples at different fermentation stages (P0, P15, P30, P45) and (**b**) a Pearson correlation loading plot.

To further highlight the overall trend of the above volatile compounds, an rPCA model was developed using these molecular signal intensities, as visualized in Figure 5.

**Figure 6:**
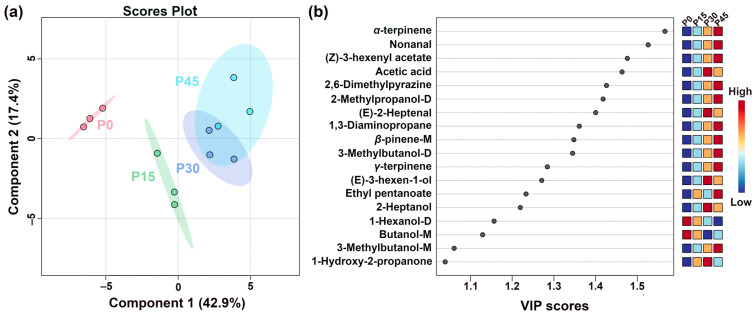
The partial least-squares discriminant analysis (PLS-DA) score plot shows the trends of clustering and separation of samples characterized by gas chromatography-ion mobility spectrometry (GC-IMS) (**a**), and the VIP score plot highlights the volatile compounds (VIP > 1.0) that are important for distinguishing the different fermentation stages (P0, P15, P30, P45) (**b**).

As is shown in Figure 5a, PC1 accounts for 86.1% of the total variance, effectively summarizing the differences between the four groups of samples along the positive and negative axes. The Pearson correlation plot of the loadings (Figure 5b) shows that P45 had higher levels of acetic acid, nonanal, *(E)*-2-heptenal, and 3-hydroxy-2-butanone, followed by P30. In contrast, P0 was characterized by higher levels of 1-pentanol-M, 1-pentanol-D, 2-methylpropanol-M, *β*-pinene-D, butanol-M, hexanal-M, 1-hexanol-M, ethyl hexanoate-M, 1-hexanol-D, 2-nonanone, styrene, decalin, and butanol-D, followed by P15. PLS-DA was further used to construct a correlation model linking volatile compound levels to sample groups, as illustrated in Figure 6a,b.

Figure 6a shows the PLS-DA scores plots of pork sour meat at different fermentation stages. The first principal component accounted for 42.9% of the variance and was the main component distinguishing the sample groups. In addition, the values of the best-fitting parameters of the PLS-DA model, namely R^2^Y, R^2^X, and Q^2^, were 0.994, 0.978, and 0.847, respectively, which indicated good predictive ability. Along Component 1, the prominent separation between P0 and P45 indicates significant differences, whereas the partial overlap between P30 and P45 suggests relative similarity. The VIP score represents a weighted sum of squared PLS-DA loadings, indicating the contribution of each variable to classification performance [26]. A screening criterion of VIP > 1.0 was used in this study to identify compounds that significantly contributed to sample classification (Figure 6b). Eighteen volatile compounds were screened as key VOCs, even those of which were alcohols. These volatile compounds are generally derived from lipid oxidation, amino acid catabolism, carbohydrate metabolism, and bacterial esterification [27]. Notably, the seven alcohols identified (e.g., 1-hexanol-D, 3-methylbutanol-D) are predominantly derived from lipid oxidation, amino acid degradation, methyl ketone reduction, and acid degradation [28]. The aldehydes, including nonanal and (E)-2-heptenal, mainly originated from lipid oxidation. Esters, such as (Z)-3-hexenyl acetate and ethyl pentanoate, which impart fruity notes, are formed from the esterification of short-chain acids and alcohols [29]. Other key VOCs included *(Z)*-3-hexenyl acetate, acetic acid, 2,6-dimethylpyriazine, 2-methypropanol-D, *(E)*-2-heptenal, 1,3-diaminopropane, *β*-pinene-M, 3-methylbutanol-D, *γ*-terpinene, *(E)*-3-hexen-1-ol, ethyl pentanoate, 2-heptanol, 1-hexanol-D, butanol-M, 3-methylbutanol-M, and 1-hydroxy-2-propanone.

#### 3.2.3. GC-MS Analysis

**Figure 7:**
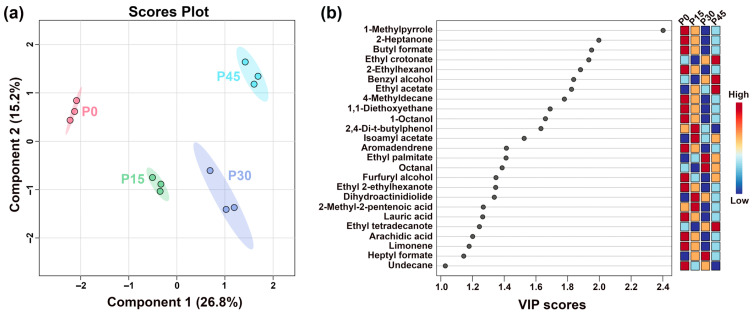
The partial least-squares discriminant analysis (PLS-DA) score plot shows the trends of clustering and separation of samples characterized by gas chromatography-mass spectrometry (GC-MS) (**a**), and the VIP score plot highlights the volatile compounds (VIP > 1.0) that are important for distinguishing the different fermentation stages (P0, P15, P30, P45) (**b**).

To further analyze differences in flavor profiles of pork sour meat at different fermentation stages, GC-MS was employed to identify and characterize volatile flavor compounds. Differences during fermentation were evaluated using PLS-DA. The best-fitting parameters of the PLS-DA model, namely R^2^Y, R^2^X, and Q^2^, were 0.992, 0.840, and 0.852, respectively, which indicated good predictive ability. As shown in Figure 7a, sour meat samples had a descending order along PC1 during fermentation. Distinct separations were found among four groups. Twenty-five volatile compounds had VIP values > 1.0 (Figure 7b). Their formation is driven by distinct microbial pathways: esters (e.g., butyl formate, ethyl crotonate, ethyl acetate) produced from microbial esterification [30]; acids (2-methyl-2-pentenoic acid and arachidic acid) derived from triglyceride hydrolysis and lipid oxidation or aldehyde and ketone transformation [31,32]; alcohols (2-ethylhexanol, benzyl alcohol, 1-octanol, furfuryl alcohol) generated from many metabolic pathways, such as lipid oxidation, amino acid metabolism, and methyl ketone reduction [33]; and aldehydes (Octanal), which are primarily derived from lipid oxidation and amino acid metabolism [34]. Notably, VIP scores of ethyl crotonate, benzyl alcohol, ethyl acetate, and ethyl tetradecanoate were higher in P45 than in P0, indicating that these compounds contributed significantly to distinguishing P45 from the others. The significantly higher VIP scores of specific compounds in P45 highlight distinct microbial enzymatic activity.

### 3.3. Bacterial Community Analysis

#### 3.3.1. Bacterial Diversity Analysis

**Figure 8:**
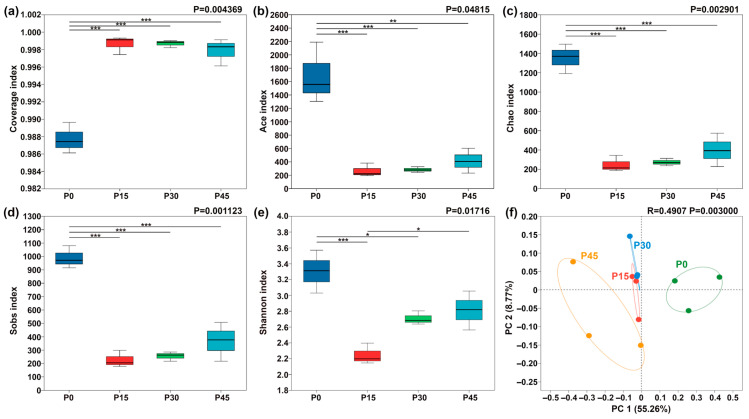
Box plots of *α*-diversity indices of bacterial communities in pork sour meat at different fermentation stages (P0, P15, P30, P45). (**a**) Coverage index; (**b**) Ace index; (**c**) Chao index; (**d**) Sobs index; (**e**) Shannon index. The symbols “*,” “**,” and “***” represent significance at *p* < 0.05, *p* < 0.01, and *p* < 0.001, respectively. Principal coordinate analysis (PCoA) of *β*-diversity in four sample groups (**f**).

Bacterial diversity in pork sour meat at different fermentation stages was assessed using *α*-diversity metrics (Figure 8). As a critical indicator of bacterial community richness and diversity, *α*-diversity indices showed significant changes over fermentation time. All samples exhibited sequencing coverage indices exceeding 0.986 (Figure 8a). The Sobs, ACE, Chao1, and Shannon indices for sour meat samples decreased sharply after 15 days and then rose slightly throughout fermentation. *β*-diversity was further assessed using PCoA, revealing clear differentiation in bacterial community composition across fermentation stages (Figure 8f). PC1 explained 55.26% of the total variation. Along PC1, P0 and P45 were positioned far apart, indicating substantial differences in community composition, whereas P15 and P30 were closer, reflecting similar bacterial community structures.

#### 3.3.2. Bacterial Taxonomic Composition Analysis

To further explore the overlap of species between different sample groups, we visualized shared and unique species using a Venn diagram.

**Figure 9:**
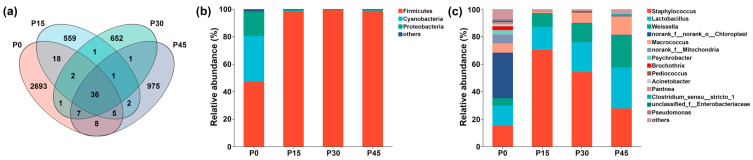
Venn diagram of four sample groups (**a**). Dynamic changes in the relative abundance of bacterial community structure in pork sour meat at different fermentation stages (P0, P15, P30, P45) at the phylum (**b**) and genus (**c**) levels during fermentation.

As shown in Figure 9a, there were 36 shared ASVs in the four fermentation stages from P0 to P45, and the number of unique ASVs decreased sharply from 2693 at P0 to 559 at P15; however, this trend reversed, with a gradual increase observed from P15 to 975 at P45. At the phylum level (Figure 9b), the microbiota comprised *Firmicutes*, *Cyanobacteria*, *Proteobacteria*, and others. Before fermentation, *Firmicutes*, *Cyanobacteria*, and *Proteobacteria* together accounted for more than 98.38% of all ASVs. After fermentation, *Firmicutes* gradually became the dominant phylum, increasing from 47.30% (P0) to 98.83% (P45). At the genus level (Figure 9c), microbial composition varied across fermentation stages. The P0 group was mainly composed of norank_f__norank_o__Chloroplast (33.23%), *Staphylococcus* (15.50%), and *Lactobacillus* (14.22%). Following fermentation, *Weissella*, *Lactobacillus*, and *Staphylococcus* became dominant. *Weissella* and *Lactobacillus* gradually increased in relative abundance over time, whereas *Staphylococcus* peaked at P15, showing an initial increase followed by a decrease. *Lactobacillus* became the most abundant genus at P45 (29.82%), followed by *Staphylococcus* (27.73%) and *Weissella* (23.77%). The relative abundance of other genera, including norank_f__norank_o__Chloroplast, norank_f__Mitochondria, Psychrobacter, and Brochothrix, was markedly reduced after fermentation.

#### 3.3.3. Bacterial Differential Analysis

**Figure 10:**
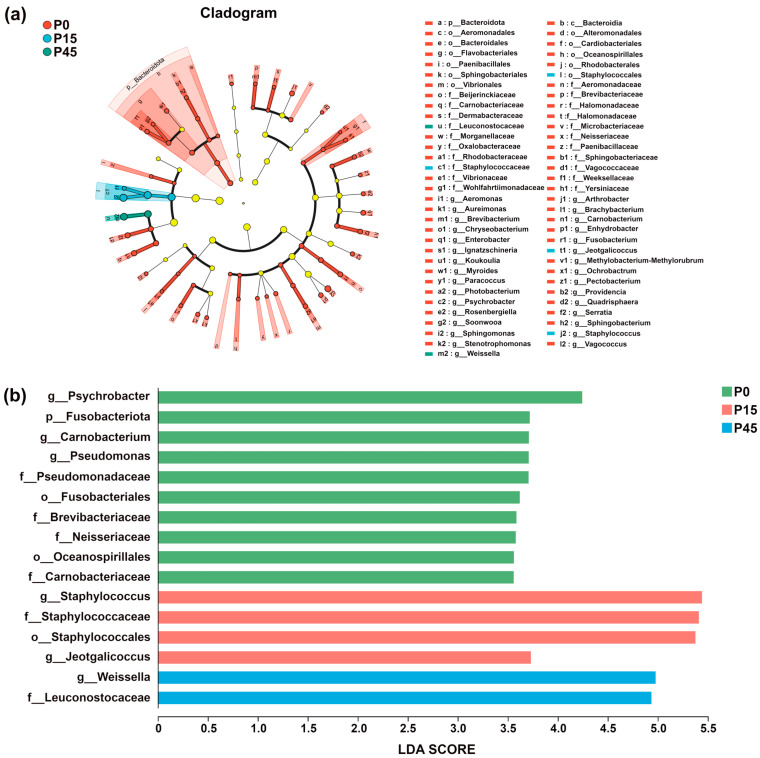
Linear discriminant analysis effect size (LEfSe) analysis plot of the bacterial community of pork sour meat at different fermentation stages (P0, P15, P30, P45). A phylogenetic cladogram showing the distribution of biomarkers across taxonomic classes (**a**). A bar diagram of linear discriminant analysis (LDA) scores for significantly differential species (LDA > 3) (**b**). Circles radiating from inside to outside represent taxonomic levels from phylum to genus. The circle diameters correspond to taxon relative abundance within respective fermentation stages. Nodes of different colors indicate microbial taxa that are significantly enriched in the corresponding group and have a significant effect on the differences between groups (*p* < 0.05). Light yellow nodes denote microorganisms that are not significantly different in this group (*p* > 0.05). The names of the species indicated by the letters in the figure are shown in the legend to the right.

Bacterial community dynamics during fermentation were analyzed using Linear Discriminant Analysis Effect Size (LEfSe) to identify significantly different bacterial taxa and assess inter-group differences [35]. Taxa with LDA scores exceeding 3.0 were selected for further analysis. In the circular representation, radiating layers correspond to taxonomic levels from phylum to genus, and circle diameters indicate relative abundance within each fermentation stage. Colored nodes represent taxa significantly enriched in a given group, contributing significantly to inter-group differences (*p* < 0.05), while light yellow nodes denote taxa without significant differences (*p* > 0.05). Species labels are shown in the figure legend. As shown in Figure 10a, the number of differential species gradually decreased during fermentation. At day 0, 59 differential species were identified, including 1 phylum and 28 genera. By day 15, diversity decreased to four characteristic taxa, primarily comprising two genera, *Jeotgalicoccus* and *Staphylococcus*. At day 45, only two differential species were observed, comprising one family (Leuconostocaceae) and one genus (*Weissella*). No significant differences were detected at day 30. LDA scores (Figure 10b) revealed clear phase-specific bacterial signatures: Psychrobacter was the most characteristic taxon in P0; *Staphylococcus* was predominant in the P15 group—consistent with its higher relative abundance in Figure 9c; and *Weissella* dominated the terminal stage (P45). The lack of significant differences in P30 suggests a transitional bacterial state during this intermediate phase.

### 3.4. Correlation Analysis of Bacterial Genera, Key Volatile Compounds, and E-Nose Sensors

**Figure 11:**
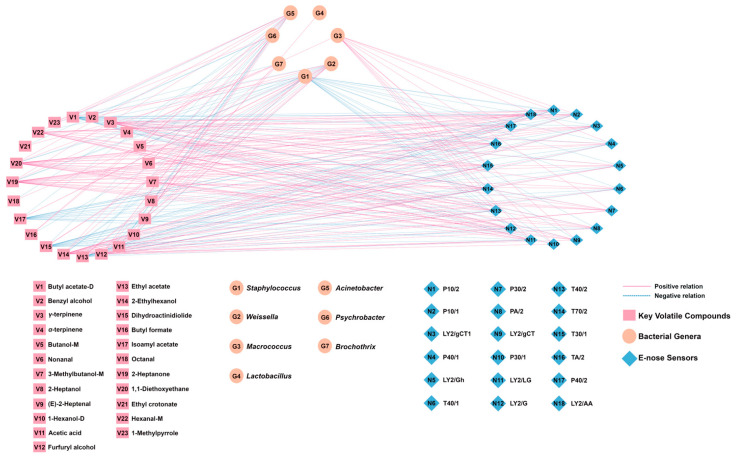
Correlation network model based on Pearson’s correlation coefficients between bacterial genera, key volatile compounds, and E-nose sensors (|r| > 0.60, *p* < 0.05, VIP > 1.0).

A correlation network model (Figure 11) was established based on the correlation coefficients between the top 7 bacterial genera, the 23 key volatile compounds, and the E-nose sensor responses. Edge connections were established using Pearson’s rank correlation coefficients meeting stringent selection criteria (|r| > 0.60, *p* < 0.05, VIP > 1.0).

As shown in Figure 11, *Weissella* exhibited significant positive correlations with nine volatile compounds. Its association with acetic acid is consistent with heterofermentative metabolism [36], and it has links to aldehydes ((*E*)-2-heptenal, octanal, nonanal) and esters (ethyl crotonate, ethyl acetate, isoamyl acetate, dihydroactinidiolide). *Staphylococcus* showed positive correlations primarily with esters (butyl acetate-D, ethyl acetate, isoamyl acetate, dihydroactinidiolide), aligning with its recognized esterase activity [37]. *Lactobacillus* also correlated with acetic acid, supporting its heterofermentative metabolism in this system [38]. Macrococcus, the fourth most abundant genus, correlated with α-terpinene and E-nose sensors for alcohols/organic solvents (P30/1, T30/1) and aromatics (T70/2). Conversely, *Psychrobacter*, *Brochothrix*, and *Acinetobacter* showed inverse correlations with key esters and acids. Although their relative abundance was <1%, potential functional roles cannot be ruled out. The E-nose sensor T70/2, which is sensitive to aromatic compounds, showed a positive correlation with six volatile compounds (γ-terpinene, hexanal-M, 2-heptanone, 2-ethylhexanol, 1,1-diethoxyethane, and furfuryl alcohol), whereas the aldehyde-sensitive E-nose sensors T40/2 and P40/2 both showed positive correlations mainly with hexanal-M, 2-ethylhexanol, 2-heptanone, and 1,1-diethoxyethane. In addition, the alcohol-sensitive E-nose sensors P30/2 and P30/1 both demonstrated strong responses with furfuryl alcohol (r = 0.737 and r = 0.754, respectively).

## 4. Discussion

Sour meat, a traditional fermented meat product popular among Chinese ethnic minority groups, is valued for its unique flavor and nutritional properties [31,39]. This study investigated the bacterial succession and changes in flavor substances in pork sour meat at different fermentation stages by using a multi-omics approach. An E-nose and an E-tongue effectively differentiated fermentation stages through aroma and taste profiles. Aroma variations detected by the E-nose may relate to consumer perception, as suggested by previous studies [30]. To further overcome the limitations of E-noses in identifying specific volatile components, GC-based analyses further identified specific volatile compounds that changed throughout the fermentation process. In this study, *(Z)*-3-hexenyl acetate, as detected by GC-IMS, exhibited the highest relative abundance among key esters at P45. Given its exceptionally low odor detection threshold in water (0.013 mg/kg), this compound probably has a positive contribution to the green, fruity notes in the overall flavor profile. Similarly, GC-MS analysis revealed ethyl crotonate, ethyl acetate, and ethyl tetradecanoate as major contributors, all of which peaked in abundance at day 45. The accumulation of esters aligns with previous studies indicating that esters formed through the esterification of alcohols and acids increase markedly during meat fermentation and play a crucial role in shaping the characteristic flavor of fermented products [28]. Therefore, it can be inferred that *(Z)*-3-hexenyl acetate, ethyl pentanoate, ethyl crotonate, ethyl acetate, and ethyl tetradecanoate collectively contribute positively to the flavor development of pork sour meat throughout fermentation. Additionally, butyl formate, a fruity ester previously reported as a key flavor component in fermented pork [40], was also identified as a significant contributor at the early stage (day 0). Its abundance progressively decreased until it became undetectable, suggesting potential microbial degradation or conversion. Future targeted metabolomics studies are needed to identify the specific degradation products and elucidate the underlying metabolic pathways.

Alcohol formation can occur through the reduction of ketones or aldehydes generated by lipid peroxidation, which have been found to play an important role in aroma formation [31]. In this study, key alcohols contributing to the flavor profile included 2-methylpropanol-D, 3-methylbutanol-D, *(E)*-3-hexen-1-ol, 2-heptanol, 1-hexanol-D, butanol-M, 3-methylbutanol-M, 2-ethylhexanol, benzyl alcohol, 1-octanol, and furfuryl alcohol. Among these, 2-methylpropanol-D, 3-methylbutanol-D, and benzyl alcohol reached their highest relative abundance in the P45 group, likely reflecting enhanced bacterial metabolism during advanced fermentation [41]. Specifically, 3-methylbutanol imparted fruity and banana-like notes, despite its relatively high odor threshold [12]. Benzyl alcohol had a floral and fruity aroma, and 2-methylpropanol-D exhibited a floral aroma. Notably, the levels of *(E)*-3-hexen-1-ol and 2-heptanol increased during early fermentation before declining. *(E)*-3-hexen-1-ol is associated with vegetal and herbaceous notes, whereas 2-heptanol had a delicate and fresh aroma, and these two substances possibly contributed to the complex flavor profiles during the early stages of fermentation.

Aldehydes, predominantly from unsaturated fatty acid oxidation [42] with minor Maillard reaction contributions, imparted characteristic fruity notes at low perception thresholds, which was especially conducive in processed meat products [43]. In the present study, nonanal was important for flavor formation and peaked in the P45 group, implying that it is related to bacterial metabolic interactions. *(E)*-2-heptenal, 2-heptanal, and octanal were found in this study, and they imparted distinctive grassy, cheesy, fruity, and sweet flavors to pork sour meat products [44].

Acids are mainly derived from lipid oxidation, phospholipid, and triglyceride hydrolysis [45]. Short-chain acids (C < 6) derived from lipid oxidation contribute significantly to the flavor of pork sour meat because of their lower thresholds and intense flavors [18]. Eight acids were identified in the present study, and six of them were short-chain acids. Among them, although lauric acid and arachidic acid were long-chain acids, they made an essential contribution to the differentiation of volatile compounds in pork sour meat at different fermentation stages. Acetic acid was higher in P30 and P45, which may be associated with the increased abundance of *Lactobacillus* and *Weissella*. Acetic acid, as a crucial volatile compound, imparted the characteristic sour taste and pungent odor of pork sour meat. 2-Methyl-2-pentenoic acid had a sour and fruity flavor. Despite its high threshold, it was identified as an important volatile compound with the ability to enable differentiation between the different fermentation stages of pork. Its formation in this study may be related to the metabolism of branched-chain amino acids by *Staphylococcus* and lipid oxidation as well as carbohydrate metabolism. *Staphylococcus* had the highest percentage in the P15 group, which is even more indicative of the relevance of 2-methyl-2-pentenoic acid to *Staphylococcus*.

Ketones in fermented meat products are primarily generated through amino acid degradation and microbial metabolism, contributing distinctive fermented and matured flavor notes [11]. In this study, 1-hydroxy-2-propanone, characterized by sweet, fruity, and mildly grassy aromas, exhibited an increase followed by a decline, peaking at day 15. 2-Heptanone, identified via GC-MS and exhibiting a high VIP score (around 2.0), was recognized as a key volatile compound distinguishing fermentation stages. However, it was only abundant at day 0 and was not detected at subsequent stages. This disappearance may reflect alterations in microbial succession and metabolic pathways during fermentation, including potential biodegradation or transformation into secondary metabolites such as alcohols or acids [46]. In this study, high levels of *α*-terpinene, *γ*-terpinene, limonene-M, and limonene-D were observed at day 45, which are derived from added spices and positively impact flavor development in fermented meats [47]. In this study, 2,6-dimethylpyrazine was identified by GC-IMS as the key volatile compound with the highest relative abundance in P45, and was able to provide a roasted flavor to the meat product [48]. The variation in 2,6-dimethylpyrazine’s relative abundance mainly originated from bacterial metabolism [49], which contributed significantly to the flavor of the pork sour meat.

Microorganisms constitute the foundation of fermented meat, profoundly shaping its sensory characteristics, nutritional quality, and safety [50]. In this study, bacterial diversity initially decreased and subsequently increased during fermentation. At the phylum level, *Firmicutes* became dominant by the late fermentation stage, comprising nearly the entire microbial community, consistent with previous findings [28]. At the genus level, *Staphylococcus*, *Lactobacillus*, and *Weissella* predominated at different fermentation stages and were identified as the principal genera, in agreement with Ma et al. [51]. In contrast to previous studies on pork sour meat [1,52], *Staphylococcus* only dominated during early to mid-fermentation, particularly on days 15 and 30. The relative abundance of *Weissella* and *Lactobacillus* increased over time. They both belonged to lactic acid bacteria (LAB) [39], enhancing the flavor and safety of pork sour meat. *Weissella* was identified as a differential species in P45, effectively discriminating late-stage fermentation. *Lactobacillus* was the second most abundant genus in most samples, consistent with Liu et al. [53]. By converting carbohydrates into organic acids, *Lactobacillus* lowers pH, inhibiting the growth of undesirable bacteria and supporting fermentation [53].

Microbial composition strongly influenced the flavor profile of pork sour meat. *Staphylococcus* played a primary role in flavor development by promoting protein degradation and lipid oxidation [54]. In this study, *Staphylococcus* was positively correlated with some esters and alcohols, consistent with previous findings [28]. *Weissella* is closely related to alcohols and hydrocarbons [39], and can also produce organic acids (acetic acid, lactic acid, etc.), contributing to flavor formation [36]. In this study, *Weissella* showed a strong positive correlation with acetic acid, despite a contradictory report [19], and was further linked to benzyl alcohol, *(E)*-2-heptenal, ethyl crotonate, octanal, ethyl acetate, isoamyl acetate, and dihydroactinidiolide. *Lactobacillus* also correlated positively with acetic acid. Notably, the high relative abundance of acetic acid in P45, coupled with its positive correlation with *Weissella*, suggests its potential role in flavor formation during late-stage fermentation.

## 5. Conclusions

This study systematically examined dynamic changes in volatile compounds and bacterial succession in pork sour meat during fermentation at 0 (P0), 15 (P15), 30 (P30), and 45 (P45) days, using a multi-analytical approach combining an E-nose, an E-tongue, GC-IMS, GC-MS, and 16S rRNA sequencing. The volatile compounds and microbial communities varied markedly across fermentation stages. GC-IMS and GC-MS identified 39 and 81 volatile compounds, respectively, encompassing esters, aldehydes, alcohols, ketones, and acids. In GC-MS, 33, 37, 54, and 52 volatile compounds were detected in the P0, P15, P30, and P45 groups, respectively. Additionally, 18 compounds (GC-IMS) and 25 compounds (GC-MS) with VIP > 1.0 were identified, serving as key discriminators of fermentation stages, with alcohols predominant in GC-IMS and esters in GC-MS. Acetic acid, nonanal, *(E)*-2-heptenal, and 3-hydroxy-2-butanone reached their highest levels after 45 days, suggesting that these compounds are key contributors to the characteristic aroma of pork sour meat products. Microbial profiling revealed *Firmicutes* as the dominant phylum, with *Staphylococcus*, *Lactobacillus*, and *Weissella* as the principal genera. From P0 to P45, the abundance of *Lactobacillus* and *Weissella* increased gradually. Pearson correlation analysis showed that *Staphylococcus*, *Lactobacillus*, and *Weissella* were primarily positively correlated with esters, alcohols, acids, and aldehydes. These findings provide baseline insights into microbial–flavor interactions that could inform future strain selection and flavor enhancement studies.

## Data Availability

The original contributions presented in this study are included in the article/Supplementary Materials. Further inquiries can be directed to the corresponding authors.

## Supplementary Materials

The following supporting information can be downloaded at: https://www.mdpi.com/article/10.3390/foods14213804/s1, Table S1: E-nose sensors and its corresponding representative sensitive compounds; Table S2: The relative content of volatile compounds in pork sour meat at different fermentation stages characterized by GC-IMS; Table S3: The relative content of volatile compounds in pork sour meat at different fermentation stages characterized by GC-MS (Mean ± SD).
