# Supplementary material for: Analysis of Changes in Flavor Profile and Bacterial Succession During Pork Fermentation Using Multi-Omics-Based Analysis
Source: Foods. 2025 Nov 6;14(21):3804. doi: 10.3390/foods14213804 (PMC12607476; doi:10.3390/foods14213804)
Supplement: Supplementary file 1 [file foods-14-03804-s001.zip › foods-3959199-supplementary.pdf]

Analysis of Changes in Flavor Profile and Bacterial Succession During Pork Fermentation Using Multi-Omics-Based Analysis

Supporting Materials

Table S1. E-nose sensors and their corresponding representative sensitive compounds.

Sensors	Performance description
LY2/LG	Sensitive to oxidizing gas (chlorine, fluorine, sulfur compounds)
LY2/G	Sensitive to ammonia and carbon monoxide (ammonia, amine compounds)
LY2/AA	Sensitive to ethanol (ethanol, ammonia)
LY2/Gh	Sensitive to ammonia/organic amines (ammonia, amine compounds)
LY2/gCT1	Sensitive to hydrogen sulfide (hydrogen sulfide)
LY2/gCT	Sensitive to propane/butane (propane, butane)
T30/1	Sensitive to organic solvents (propanol, hydrogen chloride)
P10/1	Sensitive to hydrocarbons (methane, propane, hexane (alkanes))
P10/2	Sensitive to methane (methane)
P40/1	Sensitive to fluorine (fluorine, hydrogen fluoride)
T70/2	Sensitive to aromatic compounds (benzene, toluene, xylene)
PA/2	Sensitive to ethanol and ammonia/organic amines
P30/1	Sensitive to polar compounds (ethanol)
P40/2	Sensitive to heteroatoms/chloride/aldehydes

P30/2	Sensitive to alcohol (ethanol, methanol)
T40/2	Sensitive to aldehydes (hexanal, heptanal, nonanal)
T40/1	Sensitive to chlorinated compounds (chloroform, methylene chloride)
TA/2	Sensitive to air quality

Table S2. The relative content of volatile compounds in pork sour meat at different fermentation stages as characterized by GC-IMS (Mean ± SD）.

Compounds	CAS	Formula	RI	Rt (s)	Dt (ms)	Relative content			
						P0	P15	P30	P45
**Alcohols**									
1-Hexanol-M	111-27-3	$C_6H_{14}O$	1367.2	896.965	1.32943	$2.98\times10^{-2}\pm3.18\times10^{-3}$ [a]	$1.62\times10^{-2}\pm3.11\times10^{-3}$ [b]	$1.60\times10^{-2}\pm1.66\times10^{-4}$ [b]	$1.56\times10^{-2}\pm1.25\times10^{-3}$ [b]
1-Hexanol-D	111-27-3	$C_6H_{14}O$	1365.6	892.05	1.64339	$1.44\times10^{-2}\pm3.59\times10^{-3}$ [a]	$8.75\times10^{-3}\pm1.32\times10^{-3}$ [ab]	$6.45\times10^{-3}\pm2.80\times10^{-4}$ [bc]	$5.89\times10^{-3}\pm7.56\times10^{-4}$ [c]
(E)-3-Hexen-1-ol	928-97-2	$C_6H_{12}O$	1364.5	888.773	1.56097	$5.73\times10^{-3}\pm1.55\times10^{-3}$ [a]	$5.89\times10^{-3}\pm3.97\times10^{-3}$ [a]	$1.34\times10^{-2}\pm3.41\times10^{-3}$ [a]	$1.26\times10^{-2}\pm5.37\times10^{-3}$ [a]
1-Pentanol-M	71-41-0	$C_5H_{12}O$	1254.1	597.15	1.26272	$1.24\times10^{-2}\pm1.35\times10^{-3}$ [a]	$7.42\times10^{-3}\pm1.62\times10^{-3}$ [b]	$7.89\times10^{-3}\pm3.01\times10^{-3}$ [ab]	$8.63\times10^{-3}\pm2.82\times10^{-4}$ [ab]
1-Pentanol-D	71-41-0	$C_5H_{12}O$	1254.9	598.789	1.51781	$5.99\times10^{-3}\pm1.67\times10^{-3}$ [a]	$3.53\times10^{-3}\pm1.26\times10^{-4}$ [b]	$3.37\times10^{-3}\pm4.69\times10^{-4}$ [b]	$4.17\times10^{-3}\pm4.70\times10^{-4}$ [ab]
3-Methylbutanol-M	123-51-3	$C_5H_{12}O$	1213.9	516.872	1.25094	$5.32\times10^{-2}\pm4.22\times10^{-3}$ [a]	$3.50\times10^{-2}\pm4.78\times10^{-3}$ [ab]	$3.50\times10^{-2}\pm7.53\times10^{-3}$ [b]	$4.10\times10^{-2}\pm6.10\times10^{-3}$ [ab]
3-Methylbutanol-D	123-51-3	$C_5H_{12}O$	1213	514.996	1.50543	$6.16\times10^{-2}\pm1.27\times10^{-3}$ [a]	$4.93\times10^{-2}\pm1.31\times10^{-3}$ [a]	$6.76\times10^{-2}\pm2.19\times10^{-2}$ [a]	$7.81\times10^{-2}\pm5.47\times10^{-3}$ [a]
Butanol-M	71-36-3	$C_4H_{10}O$	1150.3	423.985	1.1818	$3.91\times10^{-2}\pm6.47\times10^{-4}$ [a]	$1.97\times10^{-2}\pm1.01\times10^{-3}$ [b]	$1.66\times10^{-2}\pm2.64\times10^{-3}$ [b]	$1.79\times10^{-2}\pm8.06\times10^{-4}$ [b]
Butanol-D	71-36-3	$C_4H_{10}O$	1149.7	423.246	1.37661	$1.06\times10^{-1}\pm1.65\times10^{-2}$ [a]	$6.01\times10^{-2}\pm3.21\times10^{-3}$ [b]	$4.97\times10^{-2}\pm9.36\times10^{-3}$ [b]	$4.45\times10^{-2}\pm1.68\times10^{-3}$ [b]
2-Methylpropanol-M	78-83-1	$C_4H_{10}O$	1102.3	367.523	1.17461	$1.49\times10^{-2}\pm4.80\times10^{-4}$ [a]	$7.99\times10^{-3}\pm1.17\times10^{-3}$ [b]	$9.07\times10^{-3}\pm2.44\times10^{-3}$ [b]	$7.96\times10^{-3}\pm8.87\times10^{-4}$ [b]
2-Methylpropanol-D	78-83-1	$C_4H_{10}O$	1102.7	368.019	1.3701	$3.98\times10^{-2}\pm3.21\times10^{-3}$ [ab]	$2.66\times10^{-2}\pm4.10\times10^{-3}$ [b]	$3.40\times10^{-2}\pm1.27\times10^{-2}$ [ab]	$4.96\times10^{-2}\pm3.47\times10^{-3}$ [a]
2-Heptanol	543-49-7	$C_7H_{16}O$	1327.2	775.975	1.39724	$2.23\times10^{-3}\pm7.07\times10^{-4}$ [b]	$2.73\times10^{-3}\pm1.34\times10^{-3}$ [b]	$6.61\times10^{-3}\pm1.03\times10^{-3}$ [a]	$4.27\times10^{-3}\pm2.27\times10^{-3}$ [ab]
**Esters**									

Ethyl hexanoate-M	123-66-0	$C_8H_{16}O_2$	1241.8	572.575	1.34513	$1.67\times10^{-1}\pm1.46\times10^{-2\ a}$	$1.20\times10^{-1}\pm5.05\times10^{-3\ b}$	$9.99\times10^{-2}\pm2.38\times10^{-2\ b}$	$8.97\times10^{-2}\pm9.82\times10^{-3\ b}$
Ethyl hexanoate-D	123-66-0	$C_8H_{16}O_2$	1241	570.937	1.79448	$1.11\times10^{-1}\pm1.62\times10^{-2\ a}$	$1.08\times10^{-1}\pm1.01\times10^{-2\ a}$	$6.95\times10^{-2}\pm4.71\times10^{-2\ a}$	$6.18\times10^{-2}\pm1.56\times10^{-2\ a}$
(Z)-3-Hexenyl acetate	3681-71-8	$C_8H_{14}O_2$	1326.7	774.727	1.32807	$4.47\times10^{-3}\pm1.37\times10^{-3\ a}$	$4.83\times10^{-3}\pm1.56\times10^{-3\ a}$	$5.47\times10^{-3}\pm4.88\times10^{-4\ a}$	$7.16\times10^{-3}\pm7.30\times10^{-4\ a}$
Ethyl pentanoate	539-82-2	$C_7H_{14}O_2$	1134.4	405.257	1.30668	$5.54\times10^{-3}\pm1.44\times10^{-3\ a}$	$7.77\times10^{-3}\pm1.17\times10^{-3\ a}$	$6.52\times10^{-3}\pm8.59\times10^{-4\ a}$	$8.49\times10^{-3}\pm2.58\times10^{-3\ a}$
Butyl acetate-D	123-86-4	$C_6H_{12}O_2$	1087	354.87	1.64823	$1.95\times10^{-2}\pm4.48\times10^{-4\ b}$	$1.14\times10^{-1}\pm7.49\times10^{-2\ a}$	$6.68\times10^{-2}\pm1.93\times10^{-2\ a}$	0.05501095±0.0448076675^{a}
Butyl acetate-M	123-86-4	$C_6H_{12}O_2$	1088.2	355.614	1.26679	$6.88\times10^{-2}\pm4.79\times10^{-3\ a}$	$8.37\times10^{-2}\pm8.48\times10^{-3\ a}$	$7.26\times10^{-2}\pm1.05\times10^{-2\ a}$	$6.55\times10^{-2}\pm6.23\times10^{-3\ a}$
**Aldehydes**									
(E)-2-Heptenal	18829-55-5	$C_7H_{12}O$	1334.9	799.364	1.23944	$7.02\times10^{-3}\pm1.74\times10^{-3\ c}$	$1.82\times10^{-2}\pm6.95\times10^{-3\ b}$	$3.80\times10^{-1}\pm6.39\times10^{-3\ a}$	$3.09\times10^{-2}\pm8.47\times10^{-3\ ab}$
Heptaldehyde	111-71-7	$C_7H_{14}O$	1206.2	501.405	1.38792	$5.00\times10^{-3}\pm9.99\times10^{-4\ b}$	$1.67\times10^{-2}\pm5.34\times10^{-3\ a}$	$1.08\times10^{-2}\pm4.40\times10^{-3\ a}$	$9.68\times10^{-3}\pm1.62\times10^{-3\ ab}$
Nonanal	124-19-6	$C_9H_{18}O$	1356.7	865.166	1.43399	$6.78\times10^{-3}\pm5.21\times10^{-4\ b}$	$9.75\times10^{-3}\pm2.24\times10^{-3\ b}$	$1.70\times10^{-2}\pm2.02\times10^{-3\ a}$	$1.98\times10^{-2}\pm1.95\times10^{-3\ a}$
Hexanal-D	66-25-1	$C_6H_{12}O$	1052.3	332.303	1.55878	$3.57\times10^{-2}\pm6.07\times10^{-3\ a}$	$5.04\times10^{-2}\pm4.15\times10^{-3\ a}$	$3.51\times10^{-2}\pm1.64\times10^{-2\ a}$	$3.29\times10^{-2}\pm5.29\times10^{-3\ a}$
Hexanal-M	66-25-1	$C_6H_{12}O$	1049.7	330.57	1.25473	$4.44\times10^{-2}\pm6.28\times10^{-3\ a}$	$2.39\times10^{-2}\pm3.76\times10^{-3\ b}$	$2.39\times10^{-2}\pm8.40\times10^{-4\ b}$	$2.53\times10^{-2}\pm3.61\times10^{-3\ b}$
**Hydrocarbons**									
Styrene	100-42-5	C_8H_8	1240.2	569.299	1.44717	$7.15\times10^{-2}\pm4.75\times10^{-3\ a}$	$5.06\times10^{-2}\pm2.61\times10^{-3\ b}$	$4.46\times10^{-2}\pm1.15\times10^{-2\ b}$	$3.41\times10^{-2}\pm8.12\times10^{-3\ b}$
Decalin	91-17-8	$C_{10}H_{18}$	1150	423.616	1.26339	$8.39\times10^{-2}\pm3.61\times10^{-3\ a}$	$5.10\times10^{-2}\pm2.25\times10^{-3\ b}$	$4.67\times10^{-2}\pm5.56\times10^{-3\ b}$	$4.13\times10^{-2}\pm7.99\times10^{-3\ b}$
3-Carene	13466-78-9	$C_{10}H_{16}$	1143.5	415.976	1.67133	$3.73\times10^{-3}\pm1.62\times10^{-3\ a}$	$3.95\times10^{-3}\pm1.66\times10^{-3\ a}$	$3.47\times10^{-3}\pm1.62\times10^{-3\ a}$	$2.30\times10^{-3}\pm7.36\times10^{-4\ a}$
β-pinene-M	127-91-3	$C_{10}H_{16}$	1124.4	393.551	1.2101	$1.12\times10^{-2}\pm1.04\times10^{-3\ a}$	$8.08\times10^{-3}\pm8.62\times10^{-4\ a}$	$9.76\times10^{-3}\pm3.15\times10^{-3\ a}$	$1.09\times10^{-2}\pm1.60\times10^{-3\ a}$
β-pinene-D	127-91-3	$C_{10}H_{16}$	1124.5	393.674	1.29336	$2.11\times10^{-2}\pm3.45\times10^{-3\ a}$	$1.43\times10^{-2}\pm3.12\times10^{-3\ a}$	$1.67\times10^{-2}\pm3.29\times10^{-3\ a}$	$1.15\times10^{-2}\pm5.76\times10^{-3\ a}$
Limonene-M	138-86-3	$C_{10}H_{16}$	1200.6	490.252	1.29294	$6.79\times10^{-3}\pm3.76\times10^{-4\ b}$	$2.29\times10^{-2}\pm1.09\times10^{-3\ a}$	$1.38\times10^{-2}\pm5.48\times10^{-3\ ab}$	$9.87\times10^{-3}\pm4.52\times10^{-3\ b}$
Limonene-D	138-86-3	$C_{10}H_{16}$	1199.4	487.828	1.67542	$1.64\times10^{-2}\pm2.63\times10^{-3\ b}$	$2.94\times10^{-2}\pm2.34\times10^{-3\ a}$	$2.16\times10^{-2}\pm5.31\times10^{-3\ ab}$	$1.68\times10^{-2}\pm3.76\times10^{-3\ b}$
γ-terpinene	99-85-4	$C_{10}H_{16}$	1246	580.898	1.19978	$5.26\times10^{-3}\pm5.99\times10^{-4\ a}$	$3.34\times10^{-3}\pm2.99\times10^{-4\ b}$	$5.61\times10^{-3}\pm2.14\times10^{-3\ a}$	$5.73\times10^{-3}\pm1.22\times10^{-3\ a}$
α-terpinene	99-86-5	$C_{10}H_{16}$	1180.8	459.866	1.19657	$8.16\times10^{-3}\pm1.16\times10^{-3\ bc}$	$6.61\times10^{-3}\pm3.59\times10^{-4\ c}$	$9.97\times10^{-3}\pm1.26\times10^{-3\ ab}$	$1.15\times10^{-2}\pm9.53\times10^{-4\ a}$
**Ketones**									

2-Nonanone	821-55-6	$C_9H_{18}O$	1367.8	898.603	1.40203	$2.85\times10^{-2}\pm4.67\times10^{-3}$ ^{a}	$1.57\times10^{-2}\pm2.82\times10^{-3}$ ^{b}	$1.47\times10^{-2}\pm1.37\times10^{-3}$ ^{b}	$1.37\times10^{-2}\pm1.75\times10^{-3}$ ^{b}
2-Propanone, 1-hydroxy-	116-09-6	$C_3H_6O_2$	1301	697.089	1.20974	$7.34\times10^{-2}\pm8.05\times10^{-3}$ ^{a}	$7.96\times10^{-2}\pm1.17\times10^{-2}$ ^{a}	$7.75\times10^{-2}\pm8.43\times10^{-3}$ ^{a}	$7.00\times10^{-2}\pm2.95\times10^{-3}$ ^{a}
3-Hydroxy-2-butanone	513-86-0	$C_4H_8O_2$	1301	697.089	1.32551	$3.75\times10^{-2}\pm7.76\times10^{-3}$ ^{b}	$2.19\times10^{-1}\pm7.36\times10^{-2}$ ^{a}	$1.11\times10^{-1}\pm8.79\times10^{-2}$ ^{ab}	$1.48\times10^{-1}\pm7.95\times10^{-2}$ ^{ab}
Cyclopentanone	120-92-3	C_5H_8O	1198.2	485.403	1.36225	$8.04\times10^{-3}\pm9.94\times10^{-4}$ ^{a}	$6.97\times10^{-3}\pm1.70\times10^{-3}$ ^{a}	$6.67\times10^{-3}\pm1.12\times10^{-3}$ ^{a}	$5.37\times10^{-3}\pm7.82\times10^{-4}$ ^{a}
**Acids**									
acetic acid	64-19-7	$C_2H_4O_2$	1465.4	1193.503	1.15872	$5.80\times10^{-2}\pm9.59\times10^{-3}$ ^{b}	$9.53\times10^{-2}\pm2.59\times10^{-2}$ ^{b}	$1.72\times10^{-1}\pm2.78\times10^{-2}$ ^{a}	$1.63\times10^{-1}\pm2.19\times10^{-2}$ ^{a}
**Others**									
1,3-Diaminopropane	109-76-2	$C_3H_{10}N_2$	1357.5	867.475	1.29607	$1.81\times10^{-2}\pm2.93\times10^{-3}$ ^{a}	$1.61\times10^{-2}\pm6.40\times10^{-3}$ ^{a}	$2.67\times10^{-2}\pm4.29\times10^{-3}$ ^{a}	$2.65\times10^{-2}\pm3.81\times10^{-3}$ ^{a}
2,6-Dimethylpyrazine	108-50-9	$C_6H_8N_2$	1355.8	862.56	1.52958	$2.22\times10^{-2}\pm4.99\times10^{-3}$ ^{b}	$3.52\times10^{-2}\pm2.72\times10^{-2}$ ^{b}	$1.19\times10^{-1}\pm3.86\times10^{-2}$ ^{a}	$1.23\times10^{-1}\pm4.34\times10^{-2}$ ^{a}

* RI, RT, and DT stand for volatile compound retention index, retention time, and drift time, respectively. For each volatile compound, SEM values followed by a common superscript indicate no significant differences.

Table S3. The relative content of volatile compounds in pork sour meat at different fermentation stages as characterized by GC-MS (Mean ± SD）.

Compounds	Cas	Formula	RT	SI	RSI	Relative content			
						P0	P15	P30	P45
**Esters**									
Ethyl butanoate	105-54-4	$C_6H_{12}O_2$	3.56	918	933	$6.46\times10^{-03}\pm3.52\times10^{-03}$	$2.63\times10^{-02}\pm5.68\times10^{-03}$	$1.47\times10^{-02}\pm2.55\times10^{-03}$	$1.26\times10^{-02}\pm6.56\times10^{-03}$
Ethyl heptanoate	106-30-9	$C_9H_{18}O_2$	8.24	882	900	$1.80\times10^{-02}\pm1.44\times10^{-02}$	$1.09\times10^{-02}\pm7.30\times10^{-04}$	$1.44\times10^{-02}\pm2.50\times10^{-03}$	$1.24\times10^{-02}\pm3.76\times10^{-03}$
Ethyl caprylate	106-32-1	$C_{10}H_{20}O_2$	10.07	893	915	$1.25\times10^{-02}\pm9.62\times10^{-03}$	$1.39\times10^{-02}\pm9.07\times10^{-04}$	$2.53\times10^{-02}\pm3.62\times10^{-03}$	$2.02\times10^{-02}\pm3.96\times10^{-03}$

Ethyl laurate	106-33-2	$C_{14}H_{28}O_2$	16.99	618	663	ND	ND	ND	$1.92\times10^{-03}\pm1.20\times10^{-04}$
Methyl hexoate	106-70-7	$C_7H_{14}O_2$	5.69	909	925	ND	$3.68\times10^{-02}\pm8.24\times10^{-03}$	$2.49\times10^{-02}\pm1.54\times10^{-03}$	$1.80\times10^{-02}\pm4.47\times10^{-03}$
Ethyl isovalerate	108-64-5	$C_7H_{14}O_2$	3.93	884	885	$8.72\times10^{-03}\pm5.24\times10^{-03}$	$4.11\times10^{-02}\pm1.88\times10^{-02}$	$1.92\times10^{-02}\pm5.24\times10^{-03}$	$2.30\times10^{-02}\pm7.64\times10^{-03}$
Ethyl caprate	110-38-3	$C_{12}H_{24}O_2$	13.67	782	841	$2.44\times10^{-03}\pm9.27\times10^{-04}$	$3.11\times10^{-03}\pm1.59\times10^{-03}$	$5.42\times10^{-03}\pm1.50\times10^{-03}$	$4.95\times10^{-03}\pm2.25\times10^{-04}$
Methyl octylate	111-11-5	$C_9H_{18}O_2$	9.28	811	876	ND	$2.17\times10^{-03}\pm3.13\times10^{-04}$	$2.83\times10^{-03}\pm7.05\times10^{-04}$	$2.19\times10^{-03}\pm2.85\times10^{-04}$
Heptyl formate	112-23-2	$C_8H_{16}O_2$	10.53	748	803	ND	$3.24\times10^{-03}\pm2.89\times10^{-04}$	$3.65\times10^{-03}\pm7.29\times10^{-04}$	$2.47\times10^{-03}\pm1.20\times10^{-03}$
Methyl palmitate	112-39-0	$C_{17}H_{34}O_2$	22.29	735	846	ND	ND	$2.48\times10^{-03}\pm6.48\times10^{-04}$	$1.98\times10^{-03}\pm1.05\times10^{-04}$
Diethyl succinate	112-40-3	$C_{12}H_{26}$	4.00	798	859	$6.94\times10^{-03}\pm1.74\times10^{-03}$	ND	$3.40\times10^{-03}\pm7.16\times10^{-04}$	$4.91\times10^{-03}\pm7.63\times10^{-04}$
Ethyl nonanoate	123-29-5	$C_{11}H_{22}O_2$	11.05	735	765	$4.18\times10^{-03}\pm2.66\times10^{-03}$	$2.11\times10^{-03}\pm4.48\times10^{-04}$	$6.42\times10^{-03}\pm3.17\times10^{-03}$	$4.89\times10^{-03}\pm1.57\times10^{-03}$
Ethyl hexanoate	123-66-0	$C_8H_{16}O_2$	6.50	925	925	$2.38\times10^{-01}\pm3.28\times10^{-01}$	$5.88\times10^{-01}\pm5.04\times10^{-02}$	$4.05\times10^{-01}\pm1.57\times10^{-01}$	$2.73\times10^{-01}\pm9.79\times10^{-02}$
Isoamyl acetate	123-92-2	$C_7H_{14}O_2$	4.65	889	895	ND	$2.57\times10^{-03}\pm7.86\times10^{-04}$	$3.35\times10^{-03}\pm7.51\times10^{-04}$	$4.30\times10^{-03}\pm5.59\times10^{-04}$
Ethyl tetradecanoate	124-06-1	$C_{16}H_{32}O_2$	20.00	646	694	ND	ND	$3.25\times10^{-03}\pm7.94\times10^{-04}$	$2.85\times10^{-03}\pm6.68\times10^{-04}$
Ethyl Acetate	141-78-6	$C_4H_8O_2$	2.26	877	888	ND	$1.16\times10^{-01}\pm2.23\times10^{-02}$	$2.03\times10^{-01}\pm1.28\times10^{-01}$	$3.62\times10^{-01}\pm1.08\times10^{-01}$
Hexyl acetate	142-92-7	$C_8H_{16}O_2$	7.15	615	861	ND	$5.16\times10^{-03}\pm1.28\times10^{-03}$	ND	$5.06\times10^{-03}\pm6.14\times10^{-04}$
Dihydroactinidiolide	17092-92-1	$C_{11}H_{16}O_2$	24.17	700	810	ND	$2.71\times10^{-03}\pm5.25\times10^{-04}$	$3.74\times10^{-03}\pm3.61\times10^{-04}$	$3.62\times10^{-03}\pm1.09\times10^{-03}$
Ethyl 2-ethylhexanoate	2983-37-1	$C_{10}H_{20}O_2$	7.57	861	908	$2.25\times10^{-01}\pm2.98\times10^{-01}$	ND	ND	ND
Ethyl valerate	539-82-2	$C_7H_{14}O_2$	4.84	896	908	ND	$1.20\times10^{-02}\pm1.29\times10^{-03}$	$6.90\times10^{-03}\pm1.61\times10^{-03}$	$6.43\times10^{-03}\pm2.66\times10^{-03}$
Ethyl linoleate	544-35-4	$C_{20}H_{36}O_2$	26.80	631	669	ND	ND	$5.59\times10^{-03}\pm3.11\times10^{-03}$	$1.95\times10^{-03}\pm1.59\times10^{-04}$
Butyl formate	592-84-7	$C_5H_{10}O_2$	5.14	871	891	$1.50\times10^{-02}\pm9.86\times10^{-03}$	$4.51\times10^{-03}\pm9.35\times10^{-04}$	ND	ND
Ethyl palmitate	628-97-7	$C_{18}H_{36}O_2$	22.77	833	860	ND	$1.64\times10^{-03}\pm1.79\times10^{-04}$	$6.72\times10^{-03}\pm3.42\times10^{-03}$	$3.53\times10^{-03}\pm2.84\times10^{-03}$
Hexyl hexanoate	6378-65-0	$C_{12}H_{24}O_2$	7.97	630	722	ND	ND	$3.64\times10^{-03}\pm7.53\times10^{-05}$	ND
Ethyl 2-methylbutanoate	7452-79-1	$C_7H_{14}O_2$	3.75	933	956	$1.89\times10^{-02}\pm1.66\times10^{-02}$	$2.12\times10^{-02}\pm1.15\times10^{-02}$	$1.28\times10^{-02}\pm3.97\times10^{-03}$	$1.24\times10^{-02}\pm1.26\times10^{-03}$
Methyl acetate	79-20-9	$C_3H_6O_2$	1.96	912	957	ND	ND	$2.11\times10^{-02}\pm7.23\times10^{-03}$	$2.73\times10^{-02}\pm1.33\times10^{-02}$
Ethyl isobutyrate	97-62-1	$C_6H_{12}O_2$	2.83	768	884	ND	$3.21\times10^{-03}\pm7.55\times10^{-04}$	$1.60\times10^{-03}\pm3.06\times10^{-04}$	$2.07\times10^{-03}\pm6.66\times10^{-04}$

Ethyl crotonate	6776-19-8	$C_6H_{10}O_2$	5.36	842	872	ND	ND	$1.85\times10^{-03}\pm2.14\times10^{-04}$	$2.66\times10^{-03}\pm7.93\times10^{-04}$
**Alcohols**									
Benzyl alcohol	100-51-6	C_7H_8O	17.68	775	877	ND	ND	$3.68\times10^{-03}\pm1.54\times10^{-04}$	$4.06\times10^{-03}\pm5.17\times10^{-04}$
1-Hexanol	111-27-3	$C_6H_{14}O$	8.68	893	904	$1.86\times10^{-02}\pm1.63\times10^{-02}$	$2.45\times10^{-02}\pm4.12\times10^{-03}$	$2.53\times10^{-02}\pm4.26\times10^{-03}$	$2.29\times10^{-02}\pm5.69\times10^{-03}$
2-Ethylhexanol	104-76-7	$C_8H_{18}O$	11.12	909	937	$1.18\times10^{-02}\pm5.59\times10^{-03}$	ND	ND	ND
1-Octanol	111-87-5	$C_8H_{18}O$	12.33	827	886	$5.54\times10^{-03}\pm7.76\times10^{-04}$	ND	ND	ND
1-Nonanol	143-08-8	$C_9H_{20}O$	14.06	686	889	ND	ND	$3.34\times10^{-03}\pm1.19\times10^{-03}$	ND
4-Isopropylbenzyl Alcohol	536-60-7	$C_{10}H_{14}O$	20.84	647	764	ND	ND	ND	$1.32\times10^{-03}\pm3.59\times10^{-05}$
1-Methylcyclohexanol	590-67-0	$C_7H_{14}O$	8.15	732	811	ND	$1.36\times10^{-03}\pm6.27\times10^{-05}$	$1.53\times10^{-03}\pm1.35\times10^{-05}$	ND
4-Methyl-1-pentanol	626-89-1	$C_6H_{14}O$	7.97	721	877	ND	ND	ND	$3.51\times10^{-03}\pm1.18\times10^{-04}$
Pentanol	71-41-0	$C_5H_{12}O$	6.13	934	972	$1.02\times10^{-02}\pm5.08\times10^{-03}$	$1.13\times10^{-02}\pm2.52\times10^{-03}$	$1.82\times10^{-02}\pm1.55\times10^{-03}$	$2.28\times10^{-02}\pm8.10\times10^{-03}$
Linalool	78-70-6	$C_{10}H_{18}O$	12.10	903	907	$7.33\times10^{-03}\pm1.58\times10^{-03}$	$1.72\times10^{-02}\pm4.36\times10^{-03}$	$2.13\times10^{-02}\pm1.17\times10^{-03}$	$1.79\times10^{-02}\pm2.54\times10^{-03}$
Phenylethyl Alcohol	60-12-8	$C_8H_{10}O$	18.20	831	863	$1.14\times10^{-02}\pm1.16\times10^{-02}$	$1.13\times10^{-02}\pm3.50\times10^{-03}$	$3.17\times10^{-02}\pm1.04\times10^{-02}$	$4.35\times10^{-02}\pm1.87\times10^{-02}$
Furfuryl alcohol	98-00-0	$C_5H_6O_2$	14.25	662	906	$2.61\times10^{-03}\pm1.52\times10^{-02}$	ND	ND	ND
1-Hexadecanol, 2-methyl-	2490-48-4	$C_{17}H_{36}O$	4.27	697	753	ND	ND	$1.38\times10^{-03}\pm6.04\times10^{-04}$	ND
**Aldehydes**									
Octanal	124-13-0	$C_8H_{16}O$	7.50	853	940	ND	ND	$9.27\times10^{-03}\pm4.51\times10^{-03}$	$3.38\times10^{-03}\pm8.56\times10^{-04}$
Nonanal	124-19-6	$C_9H_{18}O$	9.39	921	924	$1.41\times10^{-02}\pm1.51\times10^{-02}$	$1.07\times10^{-02}\pm3.16\times10^{-03}$	$5.21\times10^{-02}\pm2.64\times10^{-02}$	$1.84\times10^{-02}\pm7.38\times10^{-03}$
Hexadecanal	629-80-1	$C_{16}H_{32}O$	21.22	872	926	ND	ND	$8.96\times10^{-03}\pm6.35\times10^{-04}$	$5.91\times10^{-03}\pm1.09\times10^{-03}$
Hexanal	66-25-1	$C_6H_{12}O$	4.15	692	757	$1.11\times10^{-02}\pm3.97\times10^{-03}$	$7.05\times10^{-03}\pm2.53\times10^{-03}$	ND	$1.32\times10^{-02}\pm1.13\times10^{-02}$
**Hydrocarbons**									
Aromadendrene	489-39-4	$C_{15}H_{24}$	15.06	845	875	$7.00\times10^{-03}\pm4.02\times10^{-03}$	ND	ND	ND
Limonene	138-86-3	$C_{10}H_{16}$	5.84	790	897	$1.01\times10^{-01}\pm9.40\times10^{-02}$	ND	ND	ND
2-Methylundecane	7045-71-8	$C_{12}H_{26}$	4.94	848	894	ND	ND	$2.65\times10^{-03}\pm4.78\times10^{-04}$	$2.20\times10^{-03}\pm2.15\times10^{-05}$

Pentadecane	629-62-9	$C_{15}H_{32}$	6.38	822	864	$4.49\times10^{-03}\pm2.65\times10^{-03}$	ND	ND	$1.48\times10^{-03}\pm2.47\times10^{-04}$
2,6,7-Trimethyl-decane	62108-25-2	$C_{13}H_{28}$	3.15	789	873	ND	ND	ND	$1.78\times10^{-03}\pm6.43\times10^{-04}$
Decane, 2,4,6-trimethyl-	62108-27-4	$C_{13}H_{28}$	3.41	669	891	ND	$2.23\times10^{-03}\pm1.25\times10^{-03}$	ND	ND
Hexadecane	544-76-3	$C_{16}H_{34}$	6.34	863	869	ND	ND	$2.12\times10^{-03}\pm9.53\times10^{-04}$	ND
2,6,10-Trimethyldodecane	3891-98-3	$C_{15}H_{32}$	3.40	844	877	ND	ND	$3.70\times10^{-03}\pm1.73\times10^{-03}$	ND
4-Methyldecane	2847-72-5	$C_{11}H_{24}$	3.15	721	852	$6.21\times10^{-03}\pm1.11\times10^{-03}$	$1.94\times10^{-03}\pm1.64\times10^{-04}$	ND	ND
1-Cyclopropylpentane	2511-91-3	$C_{8}H_{16}$	12.32	849	898	ND	$1.64\times10^{-03}\pm4.18\times10^{-04}$	$3.77\times10^{-03}\pm1.38\times10^{-03}$	ND
2,6,10-Trimethyltetradecane	14905-56-7	$C_{17}H_{36}$	8.53	673	770	ND	ND	$1.91\times10^{-03}\pm3.82\times10^{-04}$	ND
Dodecane	112-40-3	$C_{12}H_{26}$	4.00	798	859	$6.94\times10^{-03}\pm1.74\times10^{-03}$	ND	$3.40\times10^{-03}\pm7.16\times10^{-04}$	$4.91\times10^{-03}\pm7.63\times10^{-04}$
Undecane	1120-21-4	$C_{11}H_{24}$	3.05	635	825	$3.14\times10^{-02}\pm2.92\times10^{-02}$	ND	$3.89\times10^{-03}\pm1.98\times10^{-03}$	ND
**Ketones**									
2-Heptanone	110-43-0	$C_{7}H_{14}O$	5.67	841	863	$7.46\times10^{-03}\pm2.06\times10^{-03}$	ND	ND	ND
β-Ionone	14901-07-6	$C_{13}H_{20}O$	18.47	705	723	ND	ND	$2.45\times10^{-03}\pm7.92\times10^{-05}$	$3.04\times10^{-03}\pm1.61\times10^{-03}$
**Acids**									
Pentanoic acid	109-52-4	$C_{5}H_{10}O_{2}$	15.76	686	832	ND	$3.37\times10^{-03}\pm3.18\times10^{-03}$	ND	$2.82\times10^{-03}\pm1.58\times10^{-03}$
1-Hexanoic acid	142-62-1	$C_{6}H_{12}O_{2}$	26.69	617	816	ND	$4.03\times10^{-03}\pm1.76\times10^{-03}$	$1.53\times10^{-03}\pm6.21\times10^{-05}$	ND
Lauric acid	143-07-7	$C_{12}H_{24}O_{2}$	19.11	633	779	$1.87\times10^{-01}\pm2.60\times10^{-01}$	ND	ND	ND
2-Methyl-2-pentenoic acid	3142-72-1	$C_{6}H_{10}O_{2}$	7.97	639	714	ND	$3.41\times10^{-03}\pm3.06\times10^{-04}$	ND	ND
Arachidic acid	506-30-9	$C_{20}H_{40}O_{2}$	20.69	660	704	$1.01\times10^{-02}\pm9.25\times10^{-03}$	ND	ND	ND
Acetic acid	64-19-7	$C_{2}H_{4}O_{2}$	14.66	750	967	ND	ND	ND	$3.05\times10^{-03}\pm8.13\times10^{-04}$
5-Aminovaleric acid	660-88-8	$C_{5}H_{11}NO_{2}$	5.14	860	911	ND	ND	$4.30\times10^{-03}\pm9.57\times10^{-04}$	ND
Methoxyacetic Acid	625-45-6	$C_{3}H_{6}O_{3}$	15.78	685	984	ND	ND	ND	$5.70\times10^{-03}\pm4.71\times10^{-03}$
**Others**									
1,1-Diethoxyethane	105-57-7	$C_{6}H_{14}O_{2}$	2.26	807	929	$1.06\times10^{-02}\pm7.84\times10^{-03}$	ND	ND	ND

3-Acetylpyrrole	1072-82-8	C_6H_7NO	19.12	788	902	ND	$1.81\times10^{-03}\pm3.32\times10^{-04}$	$1.79\times10^{-03}\pm4.11\times10^{-05}$	$2.17\times10^{-03}\pm5.44\times10^{-04}$
Tetramethylpyrazine	1124-11-4	$C_8H_{12}N_2$	10.88	814	828	$7.37\times10^{-03}\pm1.08\times10^{-03}$	$2.24\times10^{-03}\pm3.96\times10^{-04}$	$3.03\times10^{-03}\pm3.80\times10^{-04}$	$3.39\times10^{-03}\pm1.84\times10^{-04}$
Estragole	140-67-0	$C_{10}H_{12}O$	16.87	658	779	ND	ND	$2.13\times10^{-03}\pm1.36\times10^{-04}$	$2.30\times10^{-03}\pm3.16\times10^{-04}$
(Methoxymethoxymethyl)benzene	31600-55-2	$C_9H_{12}O_2$	16.15	708	870	ND	$6.18\times10^{-03}\pm3.79\times10^{-05}$	$4.36\times10^{-03}\pm3.06\times10^{-03}$	$7.19\times10^{-03}\pm2.28\times10^{-03}$
2,4,5-Trimethyl-1,3-dioxolane	3299-32-9	$C_6H_{12}O_2$	2.66	822	896	ND	ND	ND	$1.41\times10^{-02}\pm1.58\times10^{-03}$
D-Mannose	3458-28-4	$C_6H_{12}O_6$	17.26	611	671	ND	ND	$1.27\times10^{-03}\pm1.03\times10^{-04}$	ND
β-lactose	5965-66-2	$C_{12}H_{22}O_{11}$	4.27	642	686	ND	ND	ND	$1.36\times10^{-03}\pm3.92\times10^{-04}$
Formohydrazide	624-84-0	CH_4N_2O	15.14	603	850	ND	ND	$3.55\times10^{-03}\pm3.22\times10^{-03}$	ND
1-Methylpyrrole	96-54-8	C_5H_7N	5.03	761	871	$5.02\times10^{-03}\pm8.86\times10^{-04}$	$2.05\times10^{-03}\pm6.23\times10^{-04}$	ND	ND
2,4-Di-t-butylphenol	96-76-4	$C_{14}H_{22}O$	23.57	761	844	$2.92\times10^{-03}\pm5.94\times10^{-04}$	$2.02\times10^{-03}\pm3.70\times10^{-04}$	$1.91\times10^{-03}\pm4.39\times10^{-04}$	ND
1,2-Dimethyl hydrazine	540-73-8	$C_2H_8N_2$	16.65	619	954	ND	ND	$1.54\times10^{-03}\pm2.34\times10^{-04}$	$1.49\times10^{-03}\pm3.39\times10^{-04}$

RT: Retention index calculated for TG-WAXMS B capillary column (30 m × 0.25 mm × 0.25 µm). ND: not detected.
